# Maternal positions and mobility during first stage labour

**DOI:** 10.1590/S1516-31802011000500015

**Published:** 2011-09-01

## Abstract

**BACKGROUND:**

It is more common for women in the developed world, and those in low-income countries giving birth in health facilities, to labour in bed. There is no evidence that this is associated with any advantage for women or babies, although it may be more convenient for staff. Observational studies have suggested that if women lie on their backs during labour this may have adverse effects on uterine contractions and impede progress in labour.

**OBJECTIVE:**

The purpose of the review is to assess the effects of encouraging women to assume different upright positions (including walking, sitting, standing and kneeling) versus recumbent positions (supine, semi-recumbent and lateral) for women in the first stage of labour on length of labour, type of delivery and other important outcomes for mothers and babies.

**CRITERIA FOR CONSIDERING STUDIES FOR THIS REVIEW:**

We searched the Cochrane Pregnancy and Childbirth Group's Trials Register (November 2008).

**SELECTION CRITERIA:**

Randomised and quasi-randomised trials comparing women randomised to upright versus recumbent positions in the first stage of labour.

**DATA COLLECTION AND ANALYSIS:**

We used methods described in the Cochrane Handbook for Systematic Reviews of Interventions for carrying out data collection, assessing study quality and analysing results. A minimum of two review authors independently assessed each study.

**MAIN RESULTS:**

The review includes 21 studies with a total of 3706 women. Overall, the first stage of labour was approximately one hour shorter for women randomised to upright as opposed to recumbent positions (MD −0.99, 95% CI −1.60 to −0.39). Women randomised to upright positions were less likely to have epidural analgesia (RR 0.83 95% CI 0.72 to 0.96). There were no differences between groups for other outcomes including length of the second stage of labour, mode of delivery, or other outcomes related to the wellbeing of mothers and babies. For women who had epidural analgesia there were no differences between those randomised to upright versus recumbent positions for any of the outcomes examined in the review. Little information on maternal satisfaction was collected, and none of the studies compared different upright or recumbent positions.

**AUTHORS’ CONCLUSIONS:**

There is evidence that walking and upright positions in the first stage of labour reduce the length of labour and do not seem to be associated with increased intervention or negative effects on mothers’ and babies’ wellbeing. Women should be encouraged to take up whatever position they find most comfortable in the first stage of labour.

**Full Professor in the Department of Gynecology, Universidade Federal de São Paulo (Unifesp), São Paulo, Brazil.**

**This is the abstract of a Cochrane Review published in the Cochrane Database of Systematic Reviews (CDSR) 2009, Issue 2, DOI:** 10.1002/14651858.CD003934.pub2 (www.thecochranelibrary.com). For full citation and authors details see reference 1.

**DOI:** 10.1002/14651858.CD004896.pub2

**For Latin America and the Caribbean, the full text is freely available from:** http://cochrane.bvsalud.org/cochrane/show.php?db=reviews&mfn=2201&id=CD003934&lang=en&dblang=&lib=COC&print=yes

## COMMENTS

This excellent systematic review,^1^ considering a total of 3706 women, compared upright versus recumbent positions during the first stage of labor.

The authors concluded there were no differences between the groups regarding the type of delivery or other outcomes relating to the wellbeing of mothers and babies. Furthermore, there was evidence that walking or remaining in an upright position during the first stage of labor reduced the length of labor.
